# Report on Rank of Medical Men in the Navy

**Published:** 1867-06

**Authors:** 


REPORT ON RANK OF MEDICAL MEN IN THE NAVY.

This paper, by Dr. Pinckney, a surgeon in the United States Navy, excited a great deal of interest and approbation. It was a most bitter but dignified protest against the discrimination made between officers of the line (military men purely) and the surgeons in the naval service in regard to title, pay, and official and social privileges. He contended that while men of the medical profession were everywhere recognized as the equals of those in other professions, in the navy they were degraded below the military, inferior in age, in experience, and rank.

He said:

Our service is overgrown with usages which sprung up in the earlier and ruder ages of naval life, and still cling to it with a power and tenacity which almost defy modern enlightenment, progress, and even law. It is probable that the national authorities which organized the existing rank of medical officers intended to confer a more substantial fact than the usages of shipboard life have permitted. Among the usages of the service is that which limits an officer's rights and comforts to the apartment in which he messes, even though his rank actually entitles him to higher privileges and greater comforts than belong to those of an inferior rank who make up the majority of the inmates of that apartment. The steerage is the most humble of those apartments, and is the dwelling-place of the very young, or those of no responsibility. The ward room gathers in it most of the commissioned and some warrant officers, and was originally occupied by none of higher rank than Lieutenant. All its usages and government are still conformed to the scale of that grade.

Now, make a medical officer in name an Admiral, and leave him to be ward room officer, and the title becomes ridiculous. It is sunk below the usages and restrictions originally designed for those of junior years and inferior rank.

There is only one mess which is superior to these restrictions, and that is the mess or messes of the commanding officers, and their associates, who may range in rank from a Lieutenant Commander to an Admiral. Sometimes there are one, sometimes two of those messes. This is very properly left to the will of the Commander-in-Chief, who may choose that he and his Captains may have one or separate establishments. The Assistant Surgeon enters the service with the rank of "Master." That this title may not be misunderstood, it may be necessary to explain that it is the lowest rank in the ward room.

For the incumbent is in modern times generally a graduate of the Naval Academy, awaiting his promotion to lieutenancy. Like the "Master," the Assistant Surgeon, at once becomes a member of the ward room mess, and unless the number of partitioned off sleeping berths contained in the ward room are occupied by his seniors, he may have the good fortune to occupy one of those that are dimly lighted by an air port, six inches in diameter. This space is so restricted, and the separation from the common apartments is so slight, that words in an ordinary voice in another become common property. After further presenting the discriminations against medical men in regard to shipboard accommodations, the Doctor said:

"The general law is that no officer shares in prize money unless his name be bourne upon the books of the vessel making the captures; but the Admiral or Commander-in-Chief has a per centage on all prizes made. The fleet Surgeon as a member of the Commander-in-Chief's staff must be with him in the flag-ship, and as a rule at the post of greatest risk, responsibility, and hazard, consequently he is not likely to have his name borne upon the books of the subordinate vessels making captures, and yet no share of prize money is allowed him."

The report suggests the following as the remedy for these evils:

1. After they have reached the rank of Commander, or are filling the position of Fleet Surgeon, let them be by right, as they often have been by courtesy, members of the cabin mess. If the mess of the Commander-in-Chief be too exalted a social position for the members of your profession who are filling the important position of Fleet Surgeon, then let them be members by right of the mess of the Commander of the ship and the Fleet Captain.

2. An equitable arrangement of prize money, most important in principle, your Committee hope to see effected. It will, however, require future legislation.

In European countries, the doctor said more liberal regulations prevail in regard to naval Surgeons than in democratic America.

The late Admiral Foote, so justly distinguished for his large medical liberality, advocated the highest rank for naval medical officers. An Admiral among the most distinguished in the service, has authorized it to be officially said that he thought the Fleet Surgeon should in our service, as in the French, be a member of the Commander-in-Chief's staff and family. We regard it as opposed to the public interests of the service, which can never be sacrificed to gross indignity without detriment.

The Committee offered the following resolution:

*Resolved*, That a Special Committee of five be appointed by the President of the Association, to present this subject before the President of the United States and the Secretary of the Navy, and urge the adoption of the changes proposed.

The resolution was adopted with a universal aye, the report having been received with prolonged applause.

When the list of Special Committees was finished, several volunteer papers were received and referred to the appropriate Sections.

The Treasurer's annual report was read, accepted, and referred.

The delegates from the several States were requested to select one of their number to sit on the Committee on Nominations. The Association then adjourned to 9 o'clock next morning.

SECOND DAY'S PROCEEDINGS.

The Association met this morning with an increased attendance, the hall being completely filled. President Askew was in the chair.

The following named persons were proposed and voted members of the Association by invitation:

Dr. J. Taylor Bradford, Augusta, Ky.

John P. Phister, Maysville, Ky.

Wm. L. Atkins, Geo. H. Whiting, A. B. Duke, M. L. Forsythe, Paul Rankin, of Kentucky.

M. W. Junkins, Galen Hart, E. P. Harrison, of Ohio.

D. R. Greenly, Meadville, Pa.

Fred. Wolf, Concord, Md.

Eugene B. Harrison, Napoleon, Ohio.

B. F. Hart, Marietta, Ohio.

Delegates from the several States presented the following named as Committeemen on nomination of officers:

Vermont—J. N. Stiles.

Massachusetts—H. R. Storer.

Rhode Island—O. Bullock.

Connecticut—B. H. Catlin.

New York—E. Elliott.

New Jersey—Samuel S. Clark.

Pennsylvania—John L. Atlee.

Delaware—H. F. Askew.

Maryland—J. J. Cockrill.
West Virginia—J. C. Hupp.
Ohio—M'Ilwaine.
Kentucky—D. W. Yandell.
Indiana—J. S. Bright.
Illinois—H. A. Johnson.
Michigan—A. B. Palmer.
Iowa—J. C. Blackburn.
Missouri—B. F. Shumard.
Texas—Hurd.
District of Columbia—Johnson Elliott.
United States Navy—N. Pinckney.
United States Army—J. J. Woodward.
Wisconsin—N. Dalton.
Kansas—John Parsons.
California—T. M. Logan.
Tennessee—W. A. Atchison.

The Secretary read a communication from Prof. Alden March, donating to the Association photographs of all the Presidents of the Association, a copy of which he transmitted to the custody of its proper officer for deposit in the archives. He also announced his pleasure to add to the collection in future. The donation was accepted with thanks.

Dr. C. C. Cox read the report of the delegation to the Foreign Medical Societies, in which he gave an interesting resume of the proceedings of the British Medical Association, which met in Chester, England, in August, 1866. Referred to the Committee on Publication.

The President of the Association appointed the following named gentlemen delegates to Foreign Medical Associations for the present year: Dr. B. Fodyce Barker, of New York; Dr. John E. Tyler, Massachusetts; Dr. Thomas C. Brinsmade, Troy, New York.

Dr. H. R. Storer arose to make an explanation. On yesterday he had made some remarks, when the report of Dr. Ray on insanity was called for and no response was made, deprecating the fact that the Superintendents of Insane Asylums held themselves aloof from this Association. His remarks, he thought, had been misunderstood, and taken in a too serious and personal light. He was happy to say that while he was then speaking, Dr. Walker, one of those Superintendents, was on his way to this meeting, with Dr. Ray's report in his pocket. He proposed that Dr. Ray be made a member of the Association by invitation, and that his report be made the special order for to-morrow morning.

Dr. Hibberd objected to departing from the rule of referring such papers to appropriate sections, as it would establish a precedent that might consume valuable time of the Association hereafter.

Dr. Walker explained that he had been delayed on his way by a railroad accident. He was surprised and grieved to learn, on arriving here, that an uncalled for assault had been made on this floor, on members of the Association of Superintendents of Insane Asylums. Every member of that Association would take it to himself, and the feeling could not be changed. He repeated the word assault, because the thing had been repeated now for the third time, and he repelled it. No member of that Association would ever again be drawn into this Association to make a personal reply. They had not thought it their duty to connect themselves with this Association, although efforts had been made to get members to come here and join the section on psychology; but if, when they came here, they were to be met with personal attack, this meeting would see the last superintendent of a Hospital for the Insane. He explained that the reason why they did not unite with this organization was, that their duties and interests as hospital superintendents were different from those of ordinary physicians. It was from no want of respect for this Association. They were, however, ready to come in as individuals, and contribute their part in the work of this Association, provided they could be received courteously.

Dr. Gross hoped the report of Dr. Ray would not be referred, but be treated with the courtesy of a hearing in open session.

Dr. Storer again disclaimed any intention to give offense. He had endeavored to satisfy Dr. Walker of this, but he did not succeed.

The report of Dr. Ray was then made the special order for this morning.

The President announced the following Committee, to lay the matter of rank and privileges of Surgeons in the U. S. Navy before the President and Secretary of the Navy.

Drs. N. S. Davis, of Illinois; J. M. Toner, District Columbia; S. D. Gross, Pennsylvania; J. J. Cockrill, Maryland; H. F. Askew, Delaware.

Dr. S. D. Gross read a report on Medical Education, which presented very much the same views as were advanced and inaugurated for practice by the convention of medical professors in session here last week. He had for twenty years been an advocate for progress in the direction of more thorough attainments, and a more rational mode of imparting instruction, beginning with the elementary and advancing to the more difficult. He condemned entirely the custom of private medical pupilage as worse than useless. The report was ordered to be printed.

Dr. Stille, President of the Medical Teachers' Convention above referred to, presented the action of that body, and asked the co-operation of the Association in carrying out the measures recommended.

Dr. Davis, Chairman of the Committee that had been appointed by this Association to call said convention, made a report, detailing the steps by which the work had been accomplished.

